# Zero-Gap Bipolar Membrane Electrolyzer for Carbon Dioxide Reduction Using Acid-Tolerant Molecular Electrocatalysts

**DOI:** 10.1021/jacs.1c13024

**Published:** 2022-04-22

**Authors:** Bhavin Siritanaratkul, Mark Forster, Francesca Greenwell, Preetam K. Sharma, Eileen H. Yu, Alexander J. Cowan

**Affiliations:** † Stephenson Institute for Renewable Energy and the Department of Chemistry, University of Liverpool, Liverpool L69 7ZF, United Kingdom; ‡ Department of Chemical Engineering, Loughborough University, Loughborough LE11 3TU, United Kingdom

## Abstract

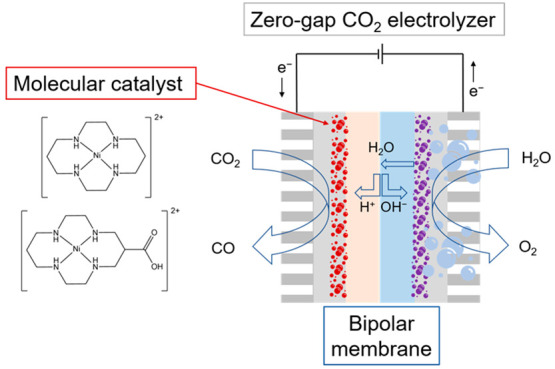

The scaling-up of electrochemical CO_2_ reduction requires circumventing the CO_2_ loss as carbonates under alkaline conditions. Zero-gap cell configurations with a reverse-bias bipolar membrane (BPM) represent a possible solution, but the catalyst layer in direct contact with the acidic environment of a BPM usually leads to H_2_ evolution dominating. Here we show that using acid-tolerant Ni molecular electrocatalysts selective (>60%) CO_2_ reduction can be achieved in a zero-gap BPM device using a pure water and CO_2_ feed. At a higher current density (100 mA cm^–2^), CO selectivity decreases, but was still >30%, due to reversible product inhibition. This study demonstrates the importance of developing acid-tolerant catalysts for use in large-scale CO_2_ reduction devices.

Electrochemical CO_2_ reduction represents a pathway to achieve a circular chemical economy by synthesizing fuels and chemicals from waste CO_2_.^1,2^ One promising configuration is a zero-gap electrolyzer, with the CO_2_ reduction catalyst loaded onto a gas-diffusion electrode (GDE) in direct contact with a cation exchange membrane (CEM), bipolar membrane (BPM), or anion exchange membrane (AEM). Zero-gap structures have been proposed as a route to high current densities and reduced manufacturing costs. Since the catalyst is in close contact with the membrane, the membrane strongly influences the local environment of the catalyst.

The majority of zero-gap studies have utilized AEM’s as a high pH limits available protons to achieve selective CO_2_ reduction vs H_2_ evolution. While this improves selectivity, CO_2_ is also “scavenged” through reaction with hydroxide to form (bi)carbonate, incurring high separation costs.^3^ In contrast, BPM and CEM devices, where the catalyst is in contact with the acidic surface of the cation exchange layer, can mitigate this issue; however, H_2_ formation dominates due to the low pH.^4^ Recent work on metal catalysts has shown that selectivity for CO_2_ reduction at lower bulk pH can be improved by a high concentration of alkali metal cations^5−7^ with liquid electrolyte GDE’s and through the use of a polymer buffering layer.^8^ Engineering the local pH shows promise, but detrimental carbonate formation within the gas diffusion layer can still occur. An alternative but yet understudied approach is to develop catalysts that are intrinsically selective toward CO_2_ reduction in acidic environments.

Few studies have explored the use of molecular catalysts on GDE’s for CO_2_ reduction, and the majority of these reports have focused on porphyrin and phthalocyanine complexes of Co and Fe at near neutral or high pH.^9,10^ To the best of our knowledge there are no past studies on the use of molecular catalyst modified GDE’s in acidic environments. Here we use molecular electrocatalysts with selectivity to CO_2_ reduction in acid environments in a zero-gap electrolyzer with a pure-water fed BPM. We used a reverse-biased BPM instead of a simpler CEM system as the sandwiched cation exchange layer/anion exchange layer (CEL/AEL) of the BPM drives water dissociation allowing the anode and cathode to be operated at different pH’s. This is beneficial as we achieve the required acidic environment at the cathode and an alkali environment at the anode, which in future studies will allow us to use earth-abundant oxygen evolution catalysts. Furthermore, studies on BPM’s in CO_2_ reduction have shown low product crossover rates.^11−13^ Hydrated CO_2_ is flowed at the cathode and deionized H_2_O is flowed at the anode. Alkaline solutions are commonly used at the anode due to the lower overpotential for oxygen evolution and higher solution conductivity. However, we used pure water which is (i) preferable for scaling up due to low corrosiveness^14,15^ and (ii) it avoids the presence of cations (e.g., K^+^) apart from H^+^ which will reach the cathode through co-ion transport,^16^ changing the local pH and complicating the analysis of the role of the molecular catalyst.

Figure 1 shows the zero-gap cell assembly using a commercial BPM (Fumasep). The molecular catalysts studied here are [Ni(Cyc)]^2+^ (Cyc = cyclam = 1,4,8,11-tetraazacyclotetradecane) and its derivative with a pendant carboxylic acid group [Ni(CycCOOH)]^2+^ (CycCOOH = 1,4,8,11-tetraazacyclotetradecane-6-carboxylic acid) which are spray coated onto carbon paper with microporous layers to form the GDE structure (at a loading of 1 mg cm^–2^ with Nafion solution as binder and ion-transporter, for details see the Supporting Information). [Ni(Cyc)]^2+^ and its derivatives have been studied extensively^17−24^ in homogeneous systems and shown to have a high CO selectivity in aqueous systems at pH 2–5, with [Ni(CycCOOH)]^2+^ being particularly active at lower pH’s.^23,25^ The selectivity at a low pH is proposed to be due to a high CO_2_ binding constant and a low p*K*_a_ of the reduced Ni^I^ center.^18,19^ There are only two prior reports of Ni cyclam-based catalysts immobilized onto a GDE,^26,27^ and one with the catalyst in a flow cell^25^ none of which used an acidic or zero-gap configuration.

**Figure 1:**
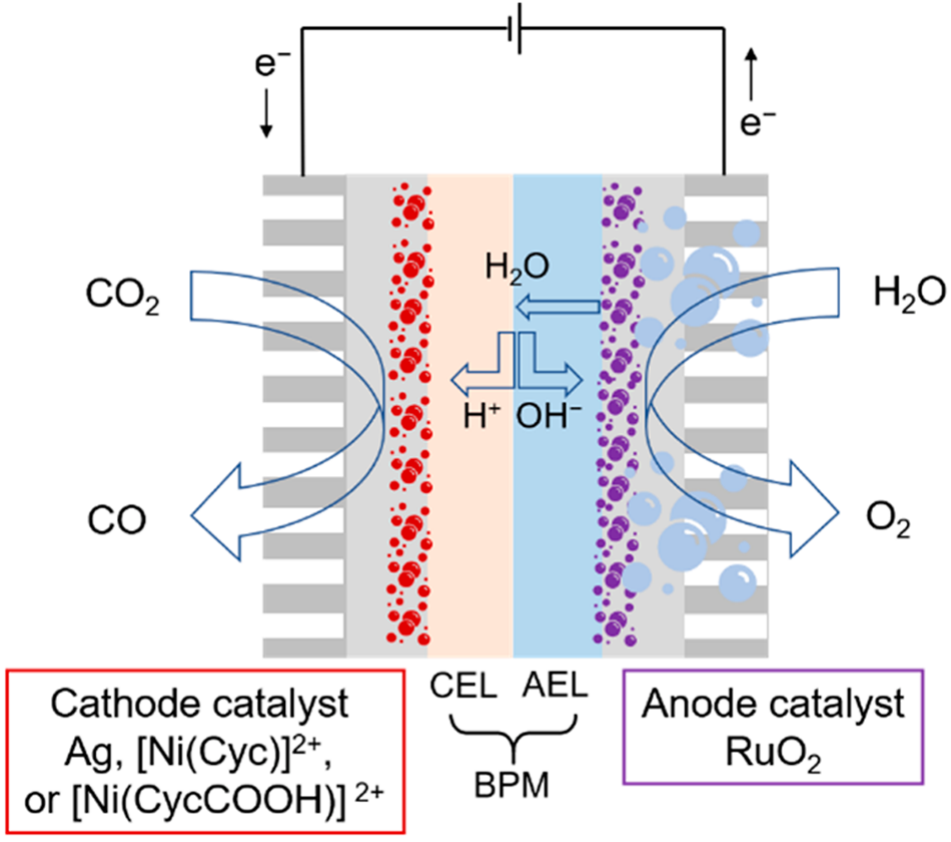
Zero-gap cell with a bipolar membrane

Figure 2A shows X-ray photoelectron spectroscopy (XPS) data from the Ni 2p region, with overlapping contributions from F Auger signals (from the polytetrafluoroethylene (PTFE) coating on the GDE and Nafion, at 835.0, 862.2, and 882.1 eV). The Ni 2p_3/2_ (856.6 eV) and Ni 2p_1/2_ (873.8 eV) peaks confirm the presence of [Ni(Cyc)]^2+^ on the GDE. The satellite peaks of Ni 2p_3/2_ at 862.3 and 866.2 eV, and Ni 2p_1/2_ at 879.4 and 883.8 eV could not be resolved in the [Ni(Cyc)]^2+^ on the GDE surface. Figures 2B–D, S1, and S2 show the scanning electron microscopy (SEM) images and corresponding Ni K_α_ and F K_α_ energy dispersive X-ray spectroscopy (EDX) elemental mapping of [Ni(Cyc)]^2+^ on a GDE. There were no visible aggregates on the carbon paper substrate and the Ni elemental mapping indicated that the complex was evenly distributed, although smaller aggregates (∼1–100 nm scale) cannot be ruled out.

**Figure 2:**
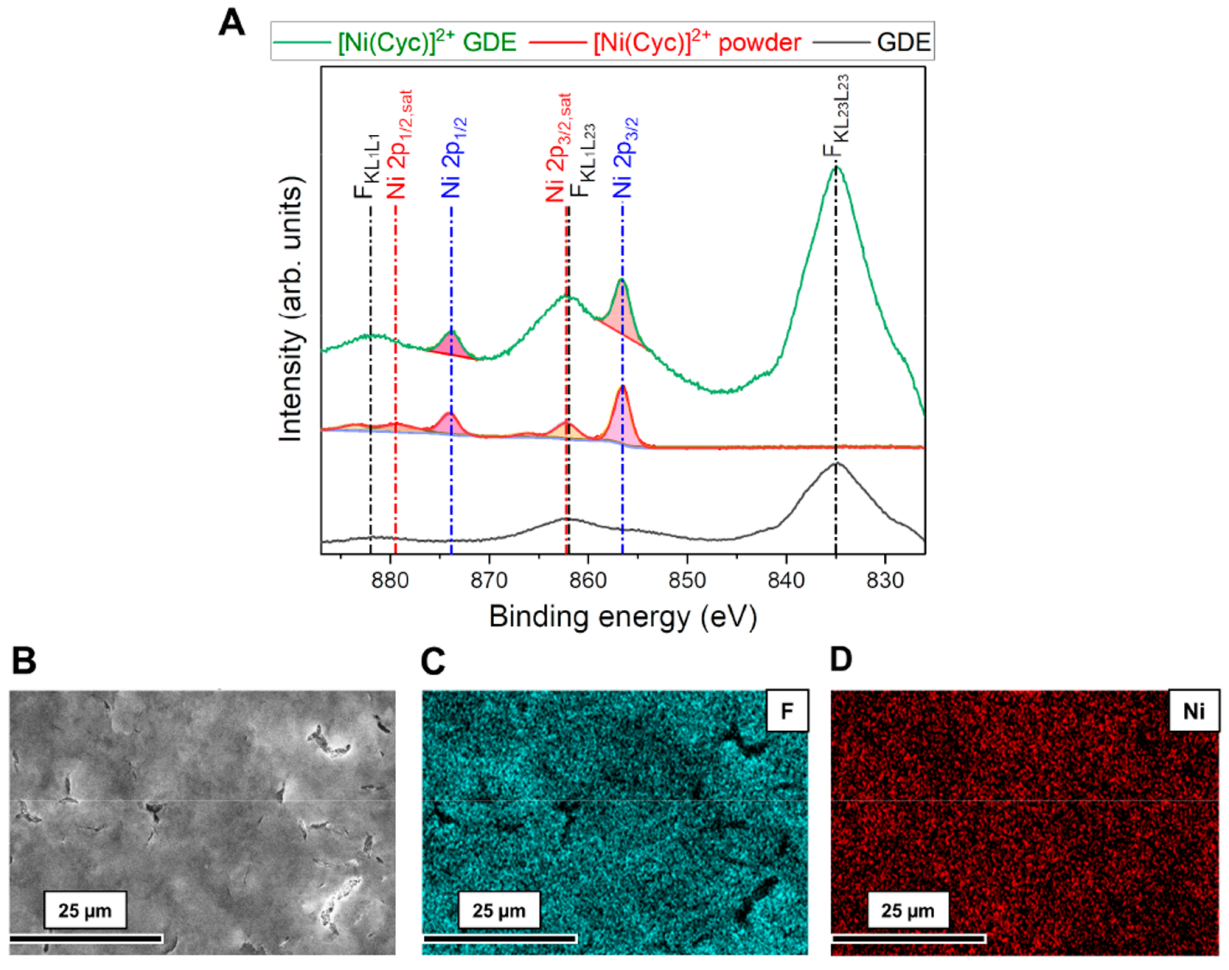
(A) XPS Ni 2p spectrum of [Ni(Cyc)]^2+^ on GDE (green), [Ni(Cyc)]^2+^ powder (red), and bare GDE (black). (B) SEM image of [Ni(Cyc)]^2+^ on GDE and corresponding (C) F K_α_ and (D) Ni K_α_ EDX maps.

We conducted chronopotentiometry of the zero-gap cell from 2.5 to 100 mA cm^–2^ for the two molecular catalysts and benchmarked against a commercial Ag nanoparticle catalyst GDE. Ag has been widely studied and is one of the most effective heterogeneous catalyst for CO production.^28−30^ The Faradaic efficiency and full cell voltages are shown in Figure 3A,B. When Ag was the cathode catalyst, H_2_ was the dominant product at all current densities, in-line with past studies of Ag with an acidic electrolyte.^4,31^ The Faradaic efficiency for CO on Ag was very low (10 ± 9%) at 12.5 mA cm^–2^ and it increased slightly with current density, reaching a maximum of 23 ± 9% at 50 mA cm^–2^. The increase in CO selectivity with current density can be explained by the expected increase in the local pH at the electrode surface.^32−34^ CO_2_ reduction (and H_2_ evolution) consumes protons, therefore the proton activity in the boundary layer of the electrolyte is lowered, decreasing H_2_ evolution.

**Figure 3:**
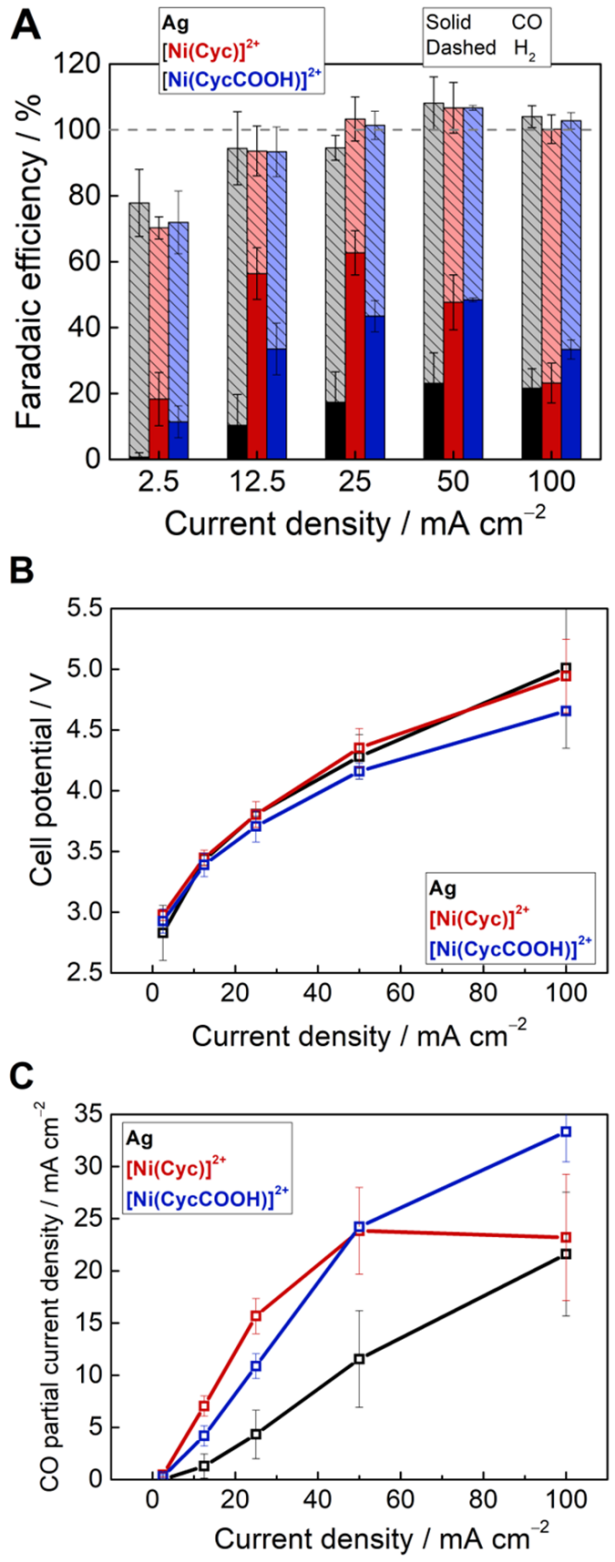
(A) Initial Faradaic efficiency for CO and H_2_, (B) full cell potential, and (C) CO partial current density at various total current densities with the cathode catalyst as Ag (black), [Ni(Cyc)]^2+^ (red), or [Ni(CycCOOH)]^2+^ (blue).

In contrast, the molecular catalysts achieved significantly higher CO Faradaic efficiency across the current range studied here. The maximum CO Faradaic efficiency reached was 63% ± 7 at 25 mA cm^−2^ for [Ni(Cyc)]^2+^ and 48% ± 1 at 50 mA cm^−2^ for [Ni(CycCOOH)]^2+^. The cell voltages were similar (∼2.8–5.0 V) across all three cathode catalysts. Although [Ni(CycCOOH)]^2+^ has been reported to achieve higher CO selectivity than [Ni(Cyc)]^2+^ on a Hg electrode in aqueous solution at a low pH,^25^ in this zero-gap configuration its selectivity was higher only at the highest current density (100 mA cm^–2^). This may be due to the different nature of the substrate (carbon paper versus Hg). Furthermore, the measured CO selectivity is the result of complex interplay between the local pH environment, intrinsic CO selectivity, and the sensitivity to CO which was not studied in previous low-current reports.

The performance of molecular catalysts in the acidic environment of our BPM cell is comparable to or exceeds recent results in the literature in which the cathode is in direct contact with the CEL side of the BPM; however, in all these cases an elevated local pH has been engineered. Yan et al. used a modified BPM designed to be near neutral (pH ∼5) on the CEL side, and reached ∼30% CO FE at ∼50 mA cm^–2^.^11^ Salvatore et al. investigated the effect of a solid-supported static buffer layer between a Ag cathode and a BPM, and reported a CO FE ∼ 10% at 100 mA cm^–2^, increasing to ∼65% with an intermediary buffer layer that would raise the local pH at the cathode.^13^

The CO partial current density (Figure 3C) shows that the activity of the molecular catalysts leveled off, especially for [Ni(Cyc)]^2+^. We estimate a lower limit for the electroactive coverage of [Ni(Cyc)]^2+^ to be 1.5 ± 0.2 × 10^–8^ mol cm^–2^ through cyclic voltammetry in acetonitrile (Figure S3). Some uncertainty remains due to different solvent penetrations from using acetonitrile, but this coverage of [Ni(Cyc)]^2+^on a GDE is 2 orders of magnitude greater than for planar electrodes.^25^ However, it is only a small fraction of the deposited catalyst (Figure S4), suggesting that future work to obtain a higher dispersion of the catalyst could enhance the CO partial current density.

We next considered the possibility of catalyst inhibition by CO formation or catalyst degradation. The change in CO selectivity with time for [Ni(Cyc)]^2+^ is shown in Figure 4A. A constant current measurement at 25 mA cm^–2^ was conducted for 1 h, then the applied current was paused for 1 h with CO_2_ and H_2_O continuing to flow, then constant 25 mA cm^–2^ was resumed for 1 h. The initial CO selectivity was 71%, which decreased to 31% after operating for 1 h. The CO selectivity recovered after the pause, but decreased again with continued operation. A similar experiment with Ag showed no appreciable change in CO:H_2_ selectivity with time, suggesting that the change is not due to local pH (Figure S5). A test with different wait times on a single sample (Figure 4B) shows that 84% of the initial CO selectivity of [Ni(Cyc)]^2+^ can be recovered after a pause of 15 min, and the extent of recovery increases with increasing pause duration.

**Figure 4:**
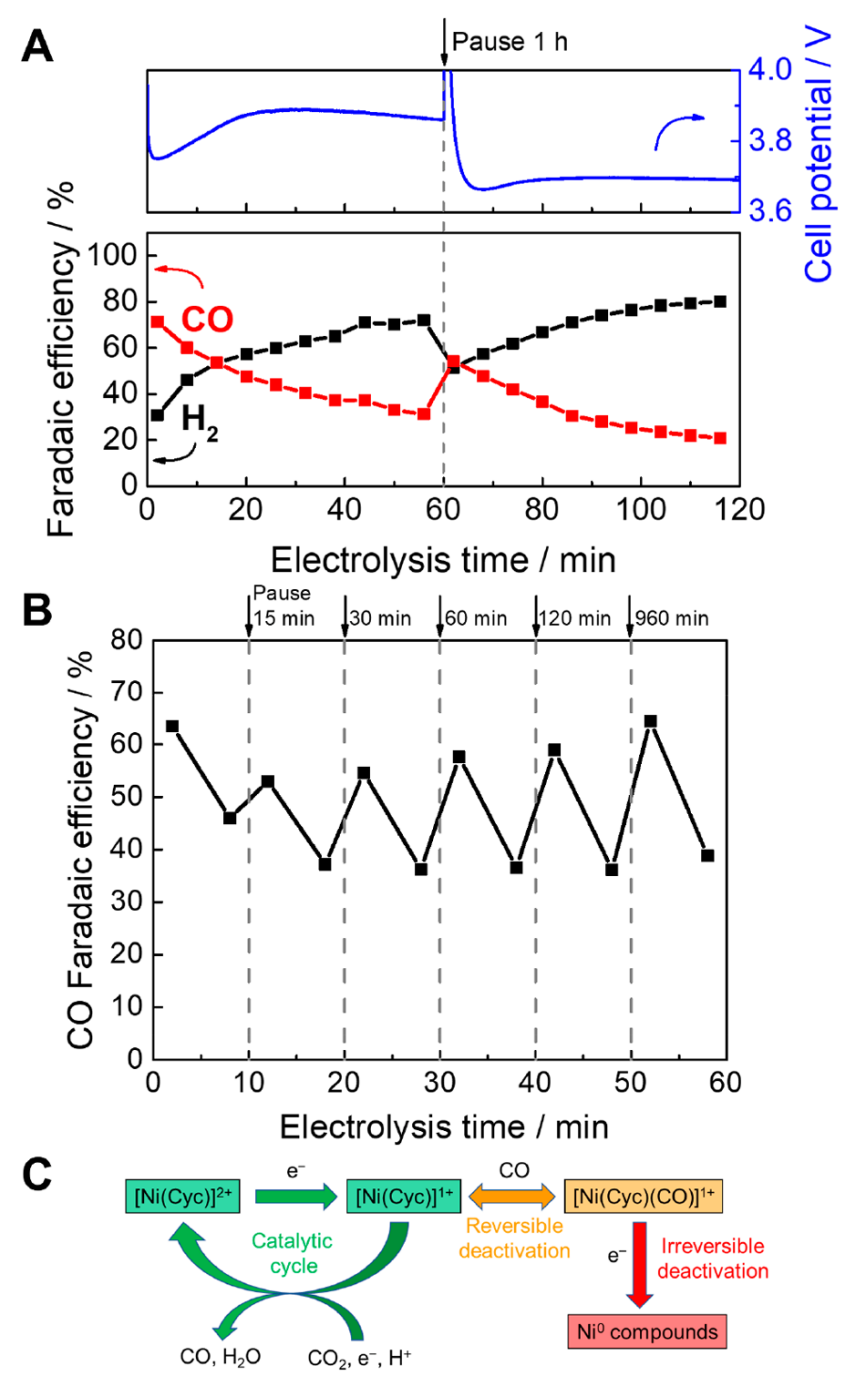
(A) Operation of [Ni(Cyc)]^2+^ in a BPM zero-gap electrolyzer at a current density of 25 mA cm^–2^ and recovery after a pause. (B) Effect of pausing electrolysis (dashed line) for different time periods on CO FE at 25 mA cm^–2^. (C) Simplified scheme of CO_2_ reduction by [Ni(Cyc)]^2+^ including reversible and irreversible deactivation pathways.

XPS and EDX postelectrolysis show a substantial loss of Ni from the GDE (Figure S6,7); however, Ni loss as the main deactivation pathway is not consistent with the recovery of selectivity. Instead, we consider inhibition of [Ni(Cyc)]^2+^ by the product CO, which can reversibly form an inactive [Ni(Cyc)(CO)]^1+^ species (Figure 4C).^35^ In an aqueous solution, the CO binding constant of Ni(Cyc)^1+^ is reported to be 4 orders of magnitude higher than its CO_2_ binding constant (7.5 × 10^5^ versus 16 M^–1^).^36−39^ Therefore, the inactive [Ni(Cyc)(CO)]^1+^ can accumulate at the CO concentrations (∼2–3%) under the highest current density in this study. Another possible concurrent deactivation/recovery pathway is the desorption and readsorption of [Ni(Cyc)]^2+^.

Since the extent of CO inhibition is proportional to the local CO concentration, the decline in selectivity can be mitigated by optimizing the reaction conditions (Figures 5 and S8). In each of these experiments, the measurement was conducted in 30 min segments with a 1 h pause in between each segment to allow the CO-inhibited species to recover. In Figure 5A (conducted in the sequence 20, 40, 80, 10, 20 sccm), the decline in CO FE decreased with increasing CO_2_ flow rate, consistent with decreased CO inhibition due to dilution by CO_2_. In Figure 5B (conducted in the sequence 25, 12.5, 100, 50, 25 mA cm^–2^), there was a general trend of faster decline in CO selectivity at higher current density, which would generate more CO. However, the trend is possibly convoluted by a further inactivation process, because a faster rate of deactivation at 25 mA cm^–2^ occurs after going to the highest currents (i.e., most reducing conditions). It is known that under very reducing conditions^40^ [Ni(Cyc)(CO)]^1+^ is irreversibly reduced to insoluble Ni^0^ compounds.^35^ Nevertheless, the results here suggest that at least part of the selectivity limitations can be overcome, for example by optimizing the reaction conditions to avoid irreversible deactivation or by pulsed operation.

**Figure 5:**
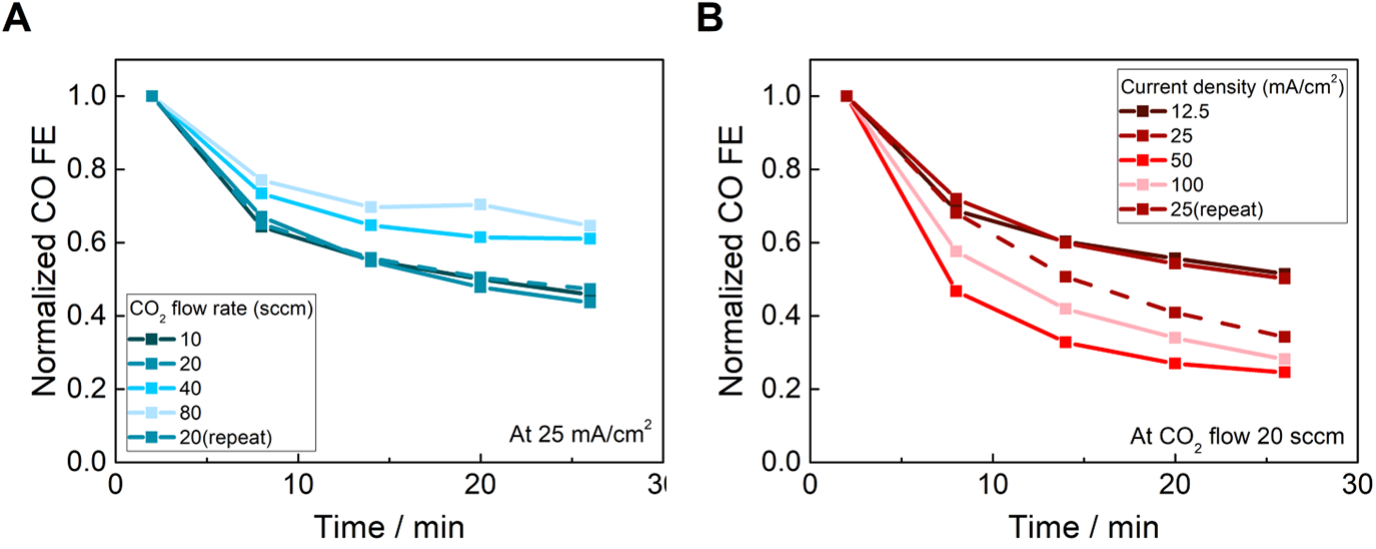
Dependence of CO Faradaic efficiency (normalized to the initial point in each segment) by [Ni(Cyc)]^2+^ on (A) CO_2_ flow rate and (B) current density.

In conclusion, the use of [Ni(Cyc)]^2+^-based molecular catalysts was demonstrated for the first time in a zero-gap CO_2_ electrolyzer with a BPM, demonstrating improved selectivity for CO_2_ reduction compared to metallic Ag catalysts up to 100 mA cm^–2^. This is a rare example of a device using only humidified CO_2_ and pure water as feedstocks. We also showed the reversible behavior of CO inhibition, only apparent at high current density, which underscores the importance of catalytic tests under realistic conditions. Our results demonstrate the viability of developing CO_2_ GDE’s that are intrinsically selective in an acidic environment.

## Supporting Information Available

Detailed experimental methods, discussion of the source of selectivity at low pH, and additional characterization and stability studies (PDF)
