# Supplementary material for: Zero-Gap Bipolar Membrane Electrolyzer for Carbon Dioxide Reduction Using Acid-Tolerant Molecular Electrocatalysts
Source: J Am Chem Soc. 2022 Apr 22;144(17):7551–6. doi: 10.1021/jacs.1c13024 (PMC9074102; doi:10.1021/jacs.1c13024)
Supplement: Supplementary file 1 — ja1c13024_si_001.pdf [file ja1c13024_si_001.pdf]

Supplementary Information

Zero-gap bipolar membrane electrolyzer for carbon dioxide reduction using acid-tolerant molecular electrocatalysts

Bhavin Siritanaratkul,[1] Mark Forster,[1] Francesca Greenwell,[1] Preetam K. Sharma,[2] Eileen H. Yu[2] and Alexander J. Cowan[1]*

1 Stephenson Institute for Renewable Energy and the Department of Chemistry, University of Liverpool, Liverpool, L69 7ZF, UK

2 Department of Chemical Engineering, Loughborough University, Loughborough, LE11 3TU, UK

1. Experimental methods

Materials

Milli-Q water (18.2 MΩ) was used throughout. CO_2 was purchased from BOC at CP grade or higher. Other reagents were used as received: Nafion N117 solution (acid form) ~5% in a mixture of lower aliphatic alcohols and water (Sigma Aldrich), Isopropyl alcohol (Sigma Aldrich), Fumasep FBM bipolar membrane (FuelCellStore, received and stored in 1 M NaCl, washed in milli-Q water for 1 h before use), carbon paper Sigracet 39 BB non-woven carbon paper with thickness of 315 µm and Microporous Layer and 5 wt% PTFE treatment (FuelCellStore), RuO_2 nanoparticles with particle size of around 5-10 nm (FuelCellStore), Ag nanoparticles with particle size of around 5-10 nm (Sigma Aldrich). $[Ni(Cyc)]^{2+}$ and $[Ni(CycCOOH)]^{2+}$ were synthesised in-house based on a previously reported procedure (see below).[1, 2]

Synthesis of $[Ni(Cyc)]Cl_2$

The cyclam ligand (1,4,8,11- tetraazacyclotetradecane) (607 mg, 3.01 mmol) was added to EtOH (100 mL) and stirred until completely dissolved. Then $NiCl_2$•$6H_2O$ (720 mg, 3.03 mmol) was added to the solution, and left stirring overnight at room temperature. Diethyl ether was added to precipitate the $[Ni(Cyc)]Cl_2$, and the precipitates were filtered, collected and dried in air.

Obtained: 817 mg, yield: 82%.

MS (ESI+) 257 [M^+-2Cl]; CHN microanalysis: Calculated for C10H24Cl2N4Ni: C, 36.40; H, 7.33; N, 16.98. Found: C, 36.39; H, 7.31; N, 16.92.

Synthesis of $[Ni(CycCOOH)]Cl_2$

$NaHCO_3$ (1.01 g, 12.0 mmol) was added to a solution of 1,4,8,11- tetraazacyclotetradecane-6-carboxylic acid (520 mg, 1.33 mmol) in water (40 mL). When gas evolution stopped, the solvent was evaporated to dryness under reduced pressure. The solid was then dissolved in ethanol (80 mL) and stirred for 40 min before and remaining solid was filtered off, leaving a clear, pale-yellow solution. The solvent was evaporated to dryness under reduced pressure overnight leaving an orange oil (303 mg, 1.24 mmol) which was dissolved in ethanol (11.6 mL). The ligand solution was then added to a solution of $NiCl_2.6H_2O$ (276 mg, 1.16 mmol) in ethanol (11.6 mL) where the solution turned dark orange upon contact. The solution was left at room temperature for 48 h where purple crystals had formed in solution. The crystals were collected and washed with ethanol and left to dry in air.

Obtained: 75 mg, yield: 17%.

MS (ESI+) 301 [M^+-2Cl]; CHN microanalysis: Calculated for C11H23Cl2N4NiO2•1.5H2O: C, 32.96; H, 6.56; N, 13.98. Found: C, 32.87; H, 6.47; N, 13.92.

Fabrication of gas diffusion electrodes

Catalysts were deposited onto Sigracet 39 BB carbon paper substrates by spray coating (Harder & Steenbeck Evolution with a N_2 stream) from a suspension. For RuO_2 anode catalyst a loading of 1 mg cm^{-2} was obtained by adding 9 mg of the nanoparticles to 1 mL H_2O and 1 mL isopropyl alcohol and 80 µL of 5% Nafion solution. The solution was sonicated for 30 min and then spray coated onto a 9 cm^2 carbon substrate placed on a hot plate at 95 ^oC. For Ag cathode catalyst a loading of 1 mg cm^{-2} was obtained by adding 5 mg of the Ag nanoparticles to 1 mL H_2O and 1 mL isopropyl alcohol and 80 µL of 5% Nafion solution. The solution was sonicated for 30 min and then spray coated onto a 5 cm^2 carbon substrate placed on a hot plate at 95 ^oC. For molecular catalysts, a catalyst loading of 1 mg cm^{-2} was obtained by adding 5 mg of the catalyst to 1 mL H_2O and 1 mL isopropyl alcohol and 80 µL of 5% Nafion solution. After fully dissolving the catalysts, the solution was sonicated for 1 min and then spray coated onto a 5 cm^2 carbon substrate placed on a hot plate at 30 ^oC.

Electrochemistry

Electrochemical measurements were carried out using a Biologic SP-200 potentiostat**.** The membrane-electrode assembly was constructed by sandwiching the bipolar membrane between the anode and cathode layers (with the cation exchange layer towards the cathode and the anion exchange layer towards the anode) and 'cold pressing' the layers together between the bipolar plates of the electrolyser cell. The membrane-electrode assembly was assembled in a 5 cm^2 electrolyser from Dioxide Materials. The electrolyser consists of a titanium anode plate with an active area of 9 cm^2 and a stainless-steel cathode plate with an active area of 5 cm^2, separated by Teflon spacers. The MEA is sandwiched between these plates to provide a zero-gap assembly to which gas is flowed to the cathode and electrolyte is flowed to the anode. The cell was assembled with a torque of 3 Nm.

CO_2 was flowed at 20 sccm, first passing through an H_2O bubbler at room temperature to humidify the gas before entering the electrolyser. Anolyte (milli-Q H_2O) was flowed across the anode at 15 ml/min. Electrochemistry was performed in a 2-electrode configuration under constant current conditions. Before measurement, the cell was pre-conditioned at open circuit, with CO_2 and anolyte flowing, for at least 30 min until the cell resistance was stabilized.

For comparison of activity at different current densities, the measurement was conducted in order of increasing currents on the same cell setup, 10 min at each current with a 30 min pause in between each segment with CO_2 and anolyte kept flowing. The faradaic efficiency reported is the initial value for each segment. The experiment was conducted in triplicate, and the error bars correspond to 1 standard deviation.

Product detection

Gaseous product in the gas outlet stream was measured by gas chromatography using a Varian CP-4900 MicroGC with a Molsieve 5Å column (10 m) with Ar carrier gas for H_2 and CO detection by a thermal conductivity detector. The measurement was carried out at constant column temperature 100 °C, constant pressure 21.7 psi, backflush time 5 s, and injection time 50 – 500 ms.

Characterization

Mass spectrometry and CHN elemental analysis was were performed by the University of Liverpool Department of Chemistry analytical services. Scanning electron microscopy (SEM) and Energy dispersive X-ray spectroscopy (EDX) were conducted with a Hitachi SEM S4800 at 20 kV. X-ray Photoelectron Spectroscopy (XPS) measurements were performed on Thermo Scientific K-Alpha X-ray Photoelectron Spectrometer using Al K_{α} source on a 400 x 400 μm^2 area. The survey scans were performed in 0-1200 eV range at 200 eV pass energy and the high-resolution scans were performed in the respective range at 50 eV pass energy. The obtained spectra were analysed using CasaXPS software (version 2.3.17). The spectra were calibrated using F 1s (689.67 eV) or N 1s (400.5 eV) peaks.

2. Note on selectivity of $[Ni(Cyc)]^{2+}$ and its derivatives

We proposed that $[Ni(Cyc)]^{2+}$-based catalysts outperform Ag in our configuration due to their acid tolerance, but we need to address an alternative explanation that $[Ni(Cyc)]^{2+}$ itself or the free cyclam ligand behaves akin to a polymer buffer layer which has been reported to increase selectivity. Polymer buffering, as reported by O'Brien et al.,[3] makes use of functional groups that can become protonated with the resultant charge hindering cation transport to the cathode. The amine groups of the free cyclam ligand can be readily protonated (pKa's 11.3, 10.2, 1.9 and 1.6)[4] which would buffer local pH in the manner suggested. However there is no free cyclam ligand on the electrode (due to the very large binding constant to Ni^{II})[5] and protonation of the amines does not occur following Ni binding to form $[Ni^{II}(Cyc)]^{2+}$.[4] This is to be expected as when coordinated to the Ni centre the N lone pair is not available and it is unable to act as a Lewis base.

If free cyclam had a positive effect on the selectivity towards CO, then the selectivity would increase with electrolysis time as the concentration of free ligand increased due to decomposition. Instead we see the opposite trend with selectivity decreasing with electrolysis time (Figure 4, 5). Additional control experiments (data not shown) with excess cyclam ligand (0.99 mg/cm^2, or 0.1 mg/cm^2) added to $[Ni(Cyc)]^{2+}$ loadings (0.1 mg/cm^2) showed a decrease in CO selectivity, table S1, confirming that excess free cyclam ligand does not play a role in achieving high CO selectivity.

Instead we highlight that $[Ni(Cyc)]^{2+}$ molecular catalysts are known to have a high selectivity for CO production in solution, including at low pH (down to pH 2 has been reported).[2, 6-13] This selectivity has been shown to arise from the high binding constant for CO_2 by the Ni^I centre, with $K_{CO2} > K_{H+}$, giving rise to the high CO:H_2 ratios observed in catalysis studies.[7, 8] The exact reason for the high K_{CO2} value is not determined experimentally but it has been proposed that for $[Ni(Cyc)]^{2+}$ the CO_2 adduct is stabilised by hydrogen bond formation with the N-H groups of the cyclam.[8] With $[Ni(CycCOOH)]^{2+}$ we proposed previously that the –COOH group may also have a role in stabilising CO_2 adducts and in facilitating their protonation further aiding selectivity.[2] In contrast Ag catalysts are known to preferentially reduced H^+ with H_2 being the dominant product at low pH.[14] Therefore we are confident that the higher CO yields in acid is due to the known high CO_2 binding constant of the $[Ni^I(Cyc)]^+$ species. A full discussion of the cause of the high CO_2 binding constant of $[Ni^I(Cyc)]^+$ catalysts is beyond the scope of this communication and refs[2, 6-13] (in particular refs[2, 12]) have already addressed this point.

Cathode	CO Faradaic efficiency / %				
	2.5 mA/cm^2	12.5 mA/cm^2	25 mA/cm^2	50 mA/cm^2	100 mA/cm^2
$[Ni(Cyc)]^{2+}$ 1 mg/cm^2 (main text results)	18	56	63	48	23
$[Ni(Cyc)]^{2+}$ 0.1 mg/cm^2	4	37	36	27	10
$[Ni(Cyc)]^{2+}$ 0.1 mg/cm^2 + free Cyc 0.1 mg/cm^2	6	26	25	17	5
$[Ni(Cyc)]^{2+}$ 0.1 mg/cm^2 + free Cyc 0.9 mg/cm^2	4	8	9	6	3
$[Ni(Cyc)]^{2+}$ 0.01 mg/cm^2	0	1	1	1	1

Table S1. Summary of the role of [Ni(Cyc)] and free Cyc ligand loading on the selectivity of the electrode in the BPM electrolyser

3. Supplementary figures

Energy Dispersive X-ray (EDX) of a $[Ni(Cyc)]^{2+}$ GDE

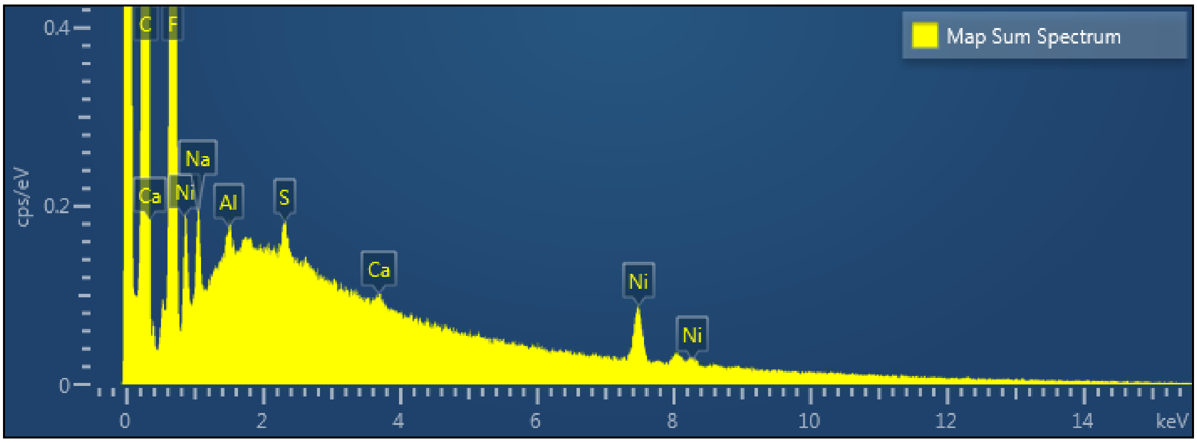

Figure S1. *EDX spectrum of a $[Ni(Cyc)]^{2+}$ GDE*

EDX of fresh $[Ni(Cyc)]^{2+}$ GDE at higher magnification

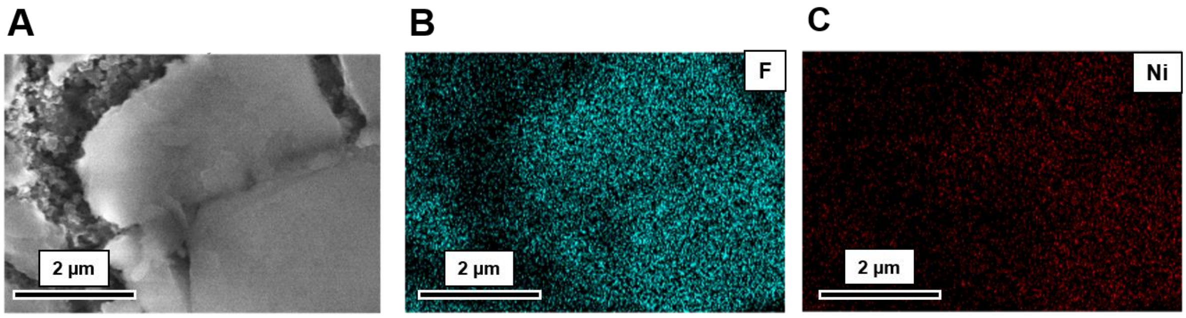

Figure S2. *(A) SEM image, and corresponding (B) F K_α and (C) Ni K_α EDX maps of a fresh $[Ni(Cyc)]^{2+}$*

Cyclic voltammetry and quantification of electroactive coverage

Using the $Ni^{3+/2+}$ couple which lies more positive of the onset for CO_2 reduction and H_2 evolution, the electroactive coverage was estimated as $1.5\pm0.2 \times 10^{-8}$ mol cm^{-2} leading to a maximum TOF of 8 ± 2 s^{-1} calculated from the highest CO partial current density of 23.2 mA cm^{-2}. Although some uncertainty remains due to different solvent penetrations from using acetonitrile, this TOF estimate is in line with previously reported TOF on glassy carbon.[1]

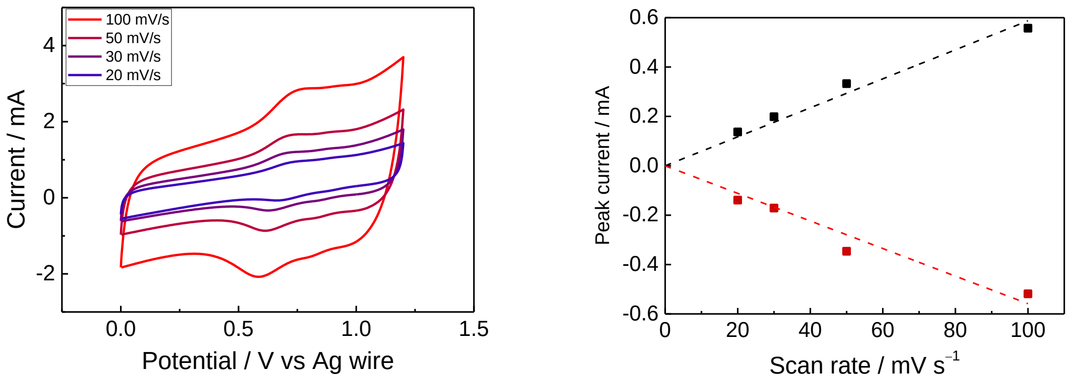

Figure S3. *Cyclic voltammetry of a $[Ni(Cyc)]^{2+}$ GDE (geometric area 1.1 cm^2) in acetonitrile with 0.1 M $TBAPF_6$ as supporting electrolyte. Counter electrode Pt mesh, Reference electrode Ag wire, room temperature, the cell was purged with Ar before measurement.*

Turnover frequency (TOF) calculation

The maximum TOF (s^{-1}) was calculated from the CO partial current density, J_{CO}, as follows:

$$TOF = \frac{J_{CO}}{nF\Gamma}$$

where n is 2, the number of electrons per CO_2-to-CO reaction, F is Faraday's constant, and Γ is the electroactive coverage of $[Ni(Cyc)]^{2+}$.

Effect of $[Ni(Cyc)]^{2+}$ loading

The estimated electroactive content when 1 mg/cm^2 of the Ni catalysts is deposited on the surface is approximately 0.5%. To study the effect of loading we have also carried out performance tests of $[Ni(Cyc)]^{2+}$ GDEs with 1/10 and 1/100 loadings (0.1 and 0.01 mg/cm^2 respectively, Figure S4). The 0.01 mg/cm^2 loading gave almost no CO production. However, the 0.1 mg/cm^2 did produce good levels of CO (FE up to 37 %) despite being only 1/10 loading. As it is still underperforming the 1 mg/cm^2 loading used throughout the manuscript we have retained the 1 mg/cm^2 loading in the main manuscript. This results in Figure S4 indicates that although a large proportion of the loaded $[Ni(Cyc)]^{2+}$ was not electroactive, a large initial loading by the current method (airbrushing onto a carbon paper substrate) was still necessary to obtain an adequate amount of electroactive catalyst and reach high performance.

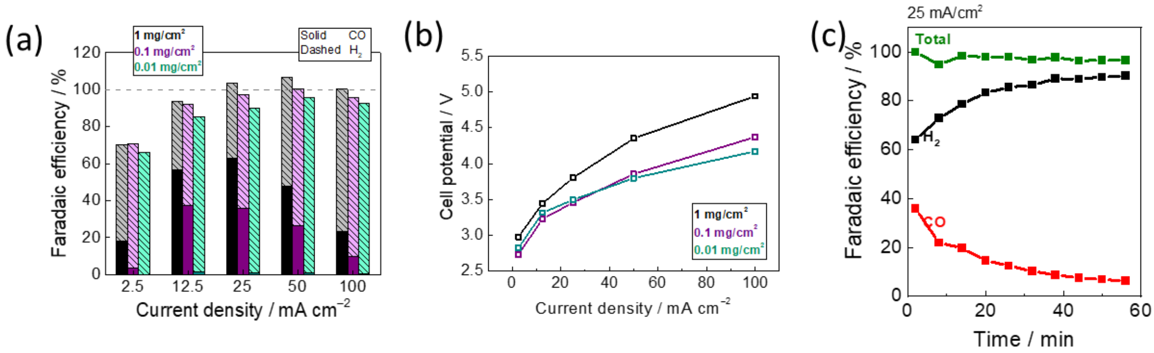

Figure S4. (a) *Faradaic efficiency and (b) full cell potentials of $[Ni(Cyc)]^{2+}$ electrodes with various loadings: 1 (black), 0.1 (purple), and 0.01 mg/cm^2 (green). (c) Shows that electrodes prepared with lower $[Ni(Cyc)]^{2+}$ loadings (0.1 mg/cm^2) at 25 mA cm^{-2} show a rapid deactivation.*

Stability of Ag

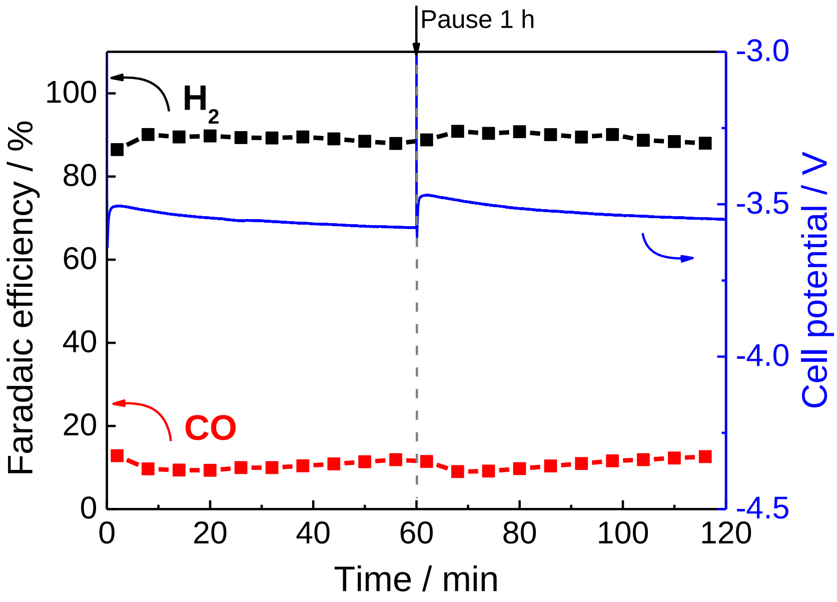

Figure S5. *Operation of Ag at a current density of 25 mAcm^{-2} and selectivity recovery after a pause. Reaction conditions: Zero-gap electrolyser cell, Fumasep bipolar membrane, cathode electrode area 5 cm^{2}, humidified CO_2 flowed across cathode at 20 ml/min, RuO_2 anode area 9 cm^{2}, pure H_2O flowed across anode at 15 ml/min.*

XPS of fresh and used $[Ni(Cyc)]^{2+}$ GDE

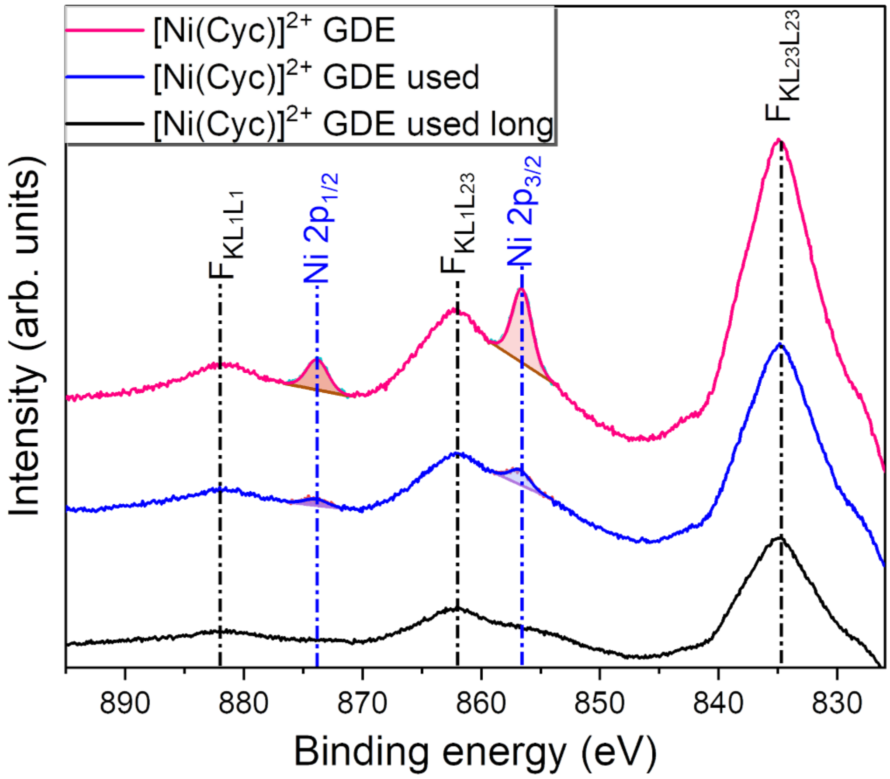

Figure S6. *XPS in the Ni 2p region for a fresh $[Ni(Cyc)]^{2+}$ GDE (red), a $[Ni(Cyc)]^{2+}$ GDE after activity measurements for 50 min (black), and a $[Ni(Cyc)]^{2+}$ GDE after operation at constant 25 mA cm^{-2} for 20 h (green).*

EDX of used $[Ni(Cyc)]^{2+}$ GDE

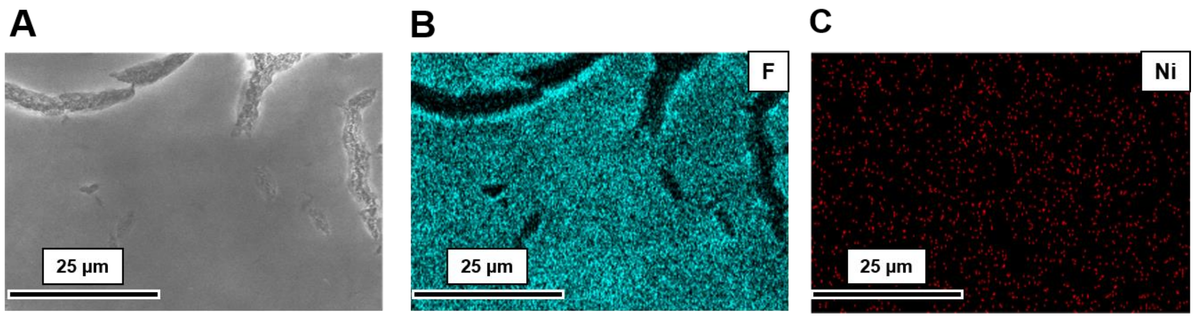

Figure S7. *(A) SEM image, and corresponding (B) F K_α and (C) Ni K_α EDX maps of a $[Ni(Cyc)]^{2+}$ GDE after activity measurements for 50 min.*

Changes in CO Faradaic efficiency under different conditions

Absolute CO Faradaic efficiency of a $[Ni(Cyc)]^{2+}$GDE corresponding to the normalized values shown in Figure 5.

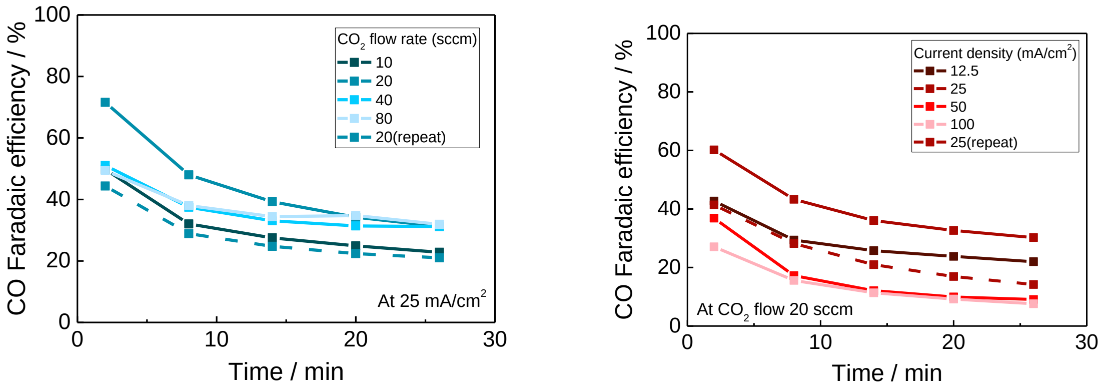

***Figure S8**. Absolute CO Faradaic efficiency with varying A) CO_2 flow rate and B) current density. Zero-gap electrolyser cell with a $[Ni(Cyc)]^{2+}$ cathode.*

References

1. Froehlich, J. D.; Kubiak, C. P., Homogeneous CO2 Reduction by Ni(cyclam) at a Glassy Carbon Electrode. *Inorg. Chem.* **2012,** *51* (7), 3932-3934.
2. Neri, G.; Aldous, I. M.; Walsh, J. J.; Hardwick, L. J.; Cowan, A. J., A highly active nickel electrocatalyst shows excellent selectivity for CO2 reduction in acidic media. *Chemical Science* **2016,** *7* (2), 1521-1526.
3. O'Brien, C. P.; Miao, R. K.; Liu, S.; Xu, Y.; Lee, G.; Robb, A.; Huang, J. E.; Xie, K.; Bertens, K.; Gabardo, C. M.; Edwards, J. P.; Dinh, C.-T.; Sargent, E. H.; Sinton, D., Single Pass CO2 Conversion Exceeding 85% in the Electrosynthesis of Multicarbon Products via Local CO2 Regeneration. *ACS Energy Letters* **2021,** *6* (8), 2952-2959.
4. Havlíčková, J.; Medová, H.; Vitha, T.; Kotek, J.; Císařová, I.; Hermann, P., Coordination properties of cyclam (1,4,8,11-tetraazacyclotetradecane) endowed with two methylphosphonic acid pendant arms in the 1,4-positions. *Dalton Transactions* **2008,** (39), 5378-5386.
5. Elias, H., Kinetics and mechanism of metal complex formation with N4-donor macrocycles of the cyclam type. *Coord. Chem. Rev.* **1999,** *187* (1), 37-73.
6. Beley, M.; Collin, J.-P.; Ruppert, R.; Sauvage, J.-P., Nickel(II)-cyclam: an extremely selective electrocatalyst for reduction of CO2 in water. *J. Chem. Soc., Chem. Commun.* **1984,** (19), 1315-1316.
7. Beley, M.; Collin, J. P.; Ruppert, R.; Sauvage, J. P., Electrocatalytic reduction of carbon dioxide by nickel cyclam2+ in water: study of the factors affecting the efficiency and the selectivity of the process. *Journal of the American Chemical Society* **1986,** *108* (24), 7461-7467.
8. Schneider, J.; Jia, H.; Kobiro, K.; Cabelli, D. E.; Muckerman, J. T.; Fujita, E., Nickel(ii) macrocycles: highly efficient electrocatalysts for the selective reduction of CO2 to CO. *Energy & Environmental Science* **2012,** *5* (11), 9502-9510.
9. Boutin, E.; Merakeb, L.; Ma, B.; Boudy, B.; Wang, M.; Bonin, J.; Anxolabéhère-Mallart, E.; Robert, M., Molecular catalysis of CO2 reduction: recent advances and perspectives in electrochemical and light-driven processes with selected Fe, Ni and Co aza macrocyclic and polypyridine complexes. *Chem. Soc. Rev.* **2020,** *49* (16), 5772-5809.
10. Jarzębińska, A.; Rowiński, P.; Zawisza, I.; Bilewicz, R.; Siegfried, L.; Kaden, T., Modified electrode surfaces for catalytic reduction of carbon dioxide. *Anal. Chim. Acta* **1999,** *396* (1), 1-12.
11. Zhanaidarova, A.; Moore, C. E.; Gembicky, M.; Kubiak, C. P., Covalent attachment of [Ni(alkynyl-cyclam)]2+ catalysts to glassy carbon electrodes. *Chem. Commun.* **2018,** *54* (33), 4116-4119.
12. Neri, G.; Walsh, J. J.; Wilson, C.; Reynal, A.; Lim, J. Y. C.; Li, X.; White, A. J. P.; Long, N. J.; Durrant, J. R.; Cowan, A. J., A functionalised nickel cyclam catalyst for CO2 reduction: electrocatalysis, semiconductor surface immobilisation and light-driven electron transfer. *Physical Chemistry Chemical Physics* **2015,** *17* (3), 1562-1566.
13. Jiang, C.; Nichols, A. W.; Walzer, J. F.; Machan, C. W., Electrochemical CO2 Reduction in a Continuous Non-Aqueous Flow Cell with [Ni(cyclam)]2+. *Inorg. Chem.* **2020,** *59* (3), 1883-1892.
14. Kim, B.; Ma, S.; Molly Jhong, H.-R.; Kenis, P. J. A., Influence of dilute feed and pH on electrochemical reduction of CO2 to CO on Ag in a continuous flow electrolyzer. *Electrochim. Acta* **2015,** *166*, 271-276.
